# Prostaglandin E_2_ Signals Through *PTGER2* to Regulate Sclerostin Expression

**DOI:** 10.1371/journal.pone.0017772

**Published:** 2011-03-16

**Authors:** Damian C. Genetos, Clare E. Yellowley, Gabriela G. Loots

**Affiliations:** 1 Department of Anatomy, Physiology, and Cell Biology, School of Veterinary Medicine, University of California Davis, Davis, California, United States of America; 2 Lawrence Livermore National Laboratory, Biology and Biotechnology Division, Livermore, California, United States of America; University of Las Palmas de Gran Canaria, Spain

## Abstract

The Wnt signaling pathway is a robust regulator of skeletal homeostasis. Gain-of-function mutations promote high bone mass, whereas loss of Lrp5 or Lrp6 co-receptors decrease bone mass. Similarly, mutations in antagonists of Wnt signaling influence skeletal integrity, in an inverse relation to Lrp receptor mutations. Loss of the Wnt antagonist Sclerostin (*Sost*) produces the generalized skeletal hyperostotic condition of sclerosteosis, which is characterized by increased bone mass and density due to hyperactive osteoblast function. Here we demonstrate that prostaglandin E_2_ (PGE_2_), a paracrine factor with pleiotropic effects on osteoblasts and osteoclasts, decreases Sclerostin expression in osteoblastic UMR106.01 cells. Decreased *Sost* expression correlates with increased expression of Wnt/TCF target genes *Axin2* and *Tcf3*. We also show that the suppressive effect of PGE_2_ is mediated through a cyclic AMP/PKA pathway. Furthermore, selective agonists for the PGE_2_ receptor EP2 mimic the effect of PGE_2_ upon *Sost*, and siRNA reduction in *Ptger2* prevents PGE_2_-induced *Sost* repression. These results indicate a functional relationship between prostaglandins and the Wnt/β-catenin signaling pathway in bone.

## Introduction

There remains considerable effort dedicated toward understanding the signaling pathways that promote skeletal anabolism. Prostaglandins (PG), such as prostaglandin E_2_ (PGE_2_), mediate osteoprogenitor proliferation [1], [2], [3] and differentiation [4], [5]. Mechanical loading *in vitro* and *in vivo* induces expression of the enzyme responsible for PG synthesis, COX-2 [6], [7], [8], whose function is required for load-induced bone formation [9], [10]. Similarly, PG administration *in vivo* increases bone mass *via* periosteal and endosteal responses [11]. Further, inhibition of PG synthesis delays fracture healing [12], [13] and promotes the formation of non-unions [14], [15], whereas localized PGE_2_ enhances bone healing [16], [17], [18], [19].

Osteoblast differentiation is also regulated by the Wnt signaling pathway [20]. Binding of Wnt ligands to a complex of Frizzled and Lrp5 or Lrp6 co-receptors promotes the stabilization of the transcription factor β-catenin, formation of a complex with TCF/LEF, and induction of Wnt target genes like *Axin2* and *Tcf3* [21], [22]. Activating mutations in *Lrp5* cause high bone mass [23], , whereas *Lrp5* deletion decreases bone mass [25], [26] and prevents load-induced bone formation [27]. Control of Wnt signaling involves sequestration of Wnts by soluble decoy receptors like sFRPs [28], [29], or Lrp5 antagonists, like Dkk1 and Sclerostin.

Deletion of the gene encoding Sclerostin (*Sost*) causes a rare sclerosing bone dysplasia, sclerosteosis (OMIM ID 269500) in both humans and murine knockout models [30], [31], [32], [33]; a related disease, van Buchem's disease (OMIM ID 239100), is caused by a distal noncoding deletion that removes regulatory elements required for the transcriptional of the *Sost* gene in adult bone [32]. Both sclerosteosis and van Buchem disease are characterized by general skeletal hyperostosis owing to hyperactive osteoblast activity. In contrast, over-expression of *Sost* causes osteopenia [34], [35] and limb deformities [36]. Mechanistically, Sclerostin was initially characterized as a BMP antagonist [34], [37], [49], but more recent reports recognize it as a potent Wnt antagonist that binds to Lrp5 and Lrp6 [38], [39], [40], [41] to increase the rate of receptor internalization [42]. Keller and Kneissel showed that PTH reduces *Sost* expression *via* PKA [43], as did Bellido *et al*. [44], and we have previously demonstrated that the regulatory element ECR5 contained within the van Buchem deletion region is necessary for bone-specific *Sost* expression in transgenic mice [35], and confers PTH responsiveness, *in vitro* [45]. Recently, we have also shown that a *Sost* null mutation partially rescues the *Lrp6^+/−^* skeletal phenotype in *Sost^−/−^*;*Lrp6^+/−^* animals [36].

Whereas both prostaglandins and Wnt signaling have parallel functions during bone anabolism, there is limited evidence for cross-talk between these two signaling pathways in pre-osteoblasts and in transformed cells. In this study, we examined the influence of PGE_2_ on Sclerostin transcription and Wnt signaling, in osteoblastic cells. We demonstrate that prostaglandin E_2_, a long-recognized regulator of osteoblast and osteoclast formation activity, decreases *Sost* expression and thereby increases Wnt signaling in osteoblastic cells. We also show that PGE_2_ transcriptional effect on *Sost* is mediated through the EP2 receptor (*Ptger2*) and cAMP, and involves mitigation of endogenous BMP and Mef2 signaling. These results attribute a novel function of prostaglandins in the regulation of Wnt signaling *via* suppression of the Wnt antagonist Sclerostin.

## Results

### Prostaglandin E_2_ decreases Sost transcription

**Figure 1:**
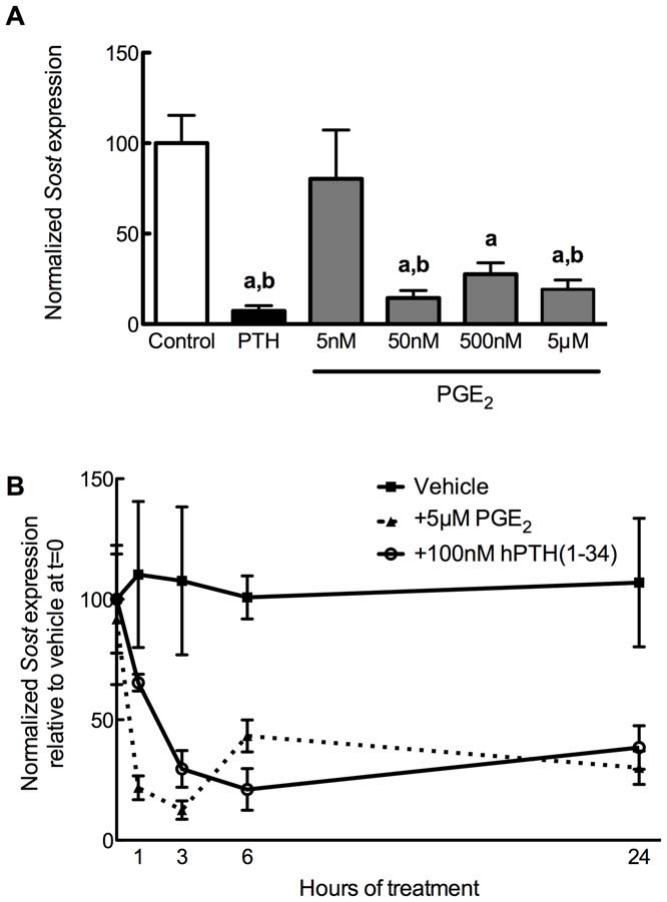
PGE_2_ decreases *Sost* expression. (**A**) Human PTH(1–34) (100 nM) or PGE_2_ (5 nM–5 µM) or vehicle control (0.05% DMSO) was added to UMR 106.01 cells for 3 hours. Total RNA was collected and analyzed for *Sost* and *Rpl32* expression by qPCR. n = 6–10 samples per treatment. **a** indicates *p*<0.05 *versus* Control; **b** indicates *P*<0.05 versus 5 nM PGE_2_. (**B**) *Sost* mRNA expression was quantified in UMR 106.01 cells after 0, 1, 2, 3, 6, or 24 hours treatment with 5 µM PGE_2_ or vehicle control. n = 4 samples per treatment. For PGE_2_, each time point is significantly different (*p*<0.05) from Control, while for PTH, every time point except 1 hr is significantly different (*p*<0.05) from Control.

Although both prostaglandins and Wnt signaling have been characterized as robust regulators of skeletal formation and homeostasis [9], [24], [46], [47], [48], there is sparse evidence whether there is direct interaction between these pathways. To that end, we first sought whether PGE_2_ demonstrated an effect upon the transcription of Sclerostin. To test this, UMR106.01 cells were chosen, as they phenotypically resemble mature osteoblasts and express high levels of *Sost* [43], [50], [51]. UMR106.01 cells were treated with 5 nM–5 µM PGE_2_ for 3 hours, after which time RNA was collected and analyzed *via* quantitative PCR (qPCR) for *Sost* expression. There was no influence of 5 nM PGE_2_ on *Sost* expression, while there was steady and progressive decrease in *Sost* levels upon 50 nM–5 µM PGE_2_ treatment (**Figure 1A**). This inhibitory effect upon Sost was not observed when cells were treated with another osteotropic prostaglandin, PGF_2α_ (5 nM–5 µM; *data not shown*). The inhibitory effect of PGE_2_ was rapid, with statistically significant suppression of *Sost* observed after one hour of treatment, and this was maintained throughout 24 hours of culture (**Figure 1B**).

### Prostaglandin E_2_ influences Wnt signaling without affecting Dkk1

**Figure 2:**
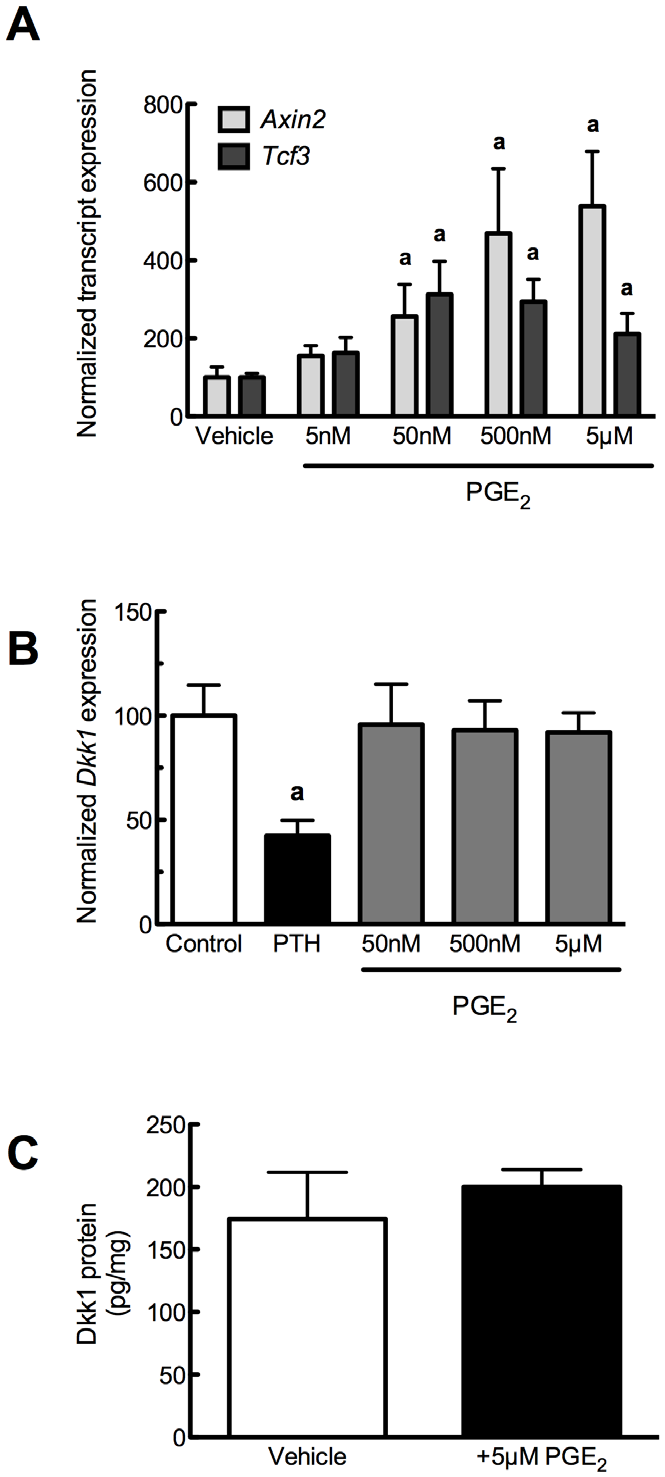
PGE_2_ increases Wnt signaling without affecting *Dkk1*. (**A**) PGE_2_ (5 nM–5 µM) or vehicle control (0.05% DMSO) was added to UMR 106.01 cells for 24 hours. Total RNA was collected and analyzed for *Axin2*, *Tcf3*, and *Rpl32* expression by qPCR. n = 4 samples. Compared to vehicle control, **a** indicates *p*<0.05. (**B**) Human PTH(1–34) (100 nM) or PGE_2_ (50 nM–5 µM) or vehicle control (0.05% DMSO) was added to UMR 106.01 cells for 3 hours. Total RNA was collected and analyzed for *Dkk1* and *Rpl32* expression by qPCR. n = 8 samples. Compared to vehicle control, **a** indicates *p*<0.05.

Functional decrease in the expression of the Wnt antagonist *Sost* should effectively increase markers of β-catenin/TCF signaling, such as *Axin2* and *Tcf3*. To that end, we observed that PGE_2_, in the same dosing range that decreased *Sost*, significantly increased *Axin2* and *Tcf3* expression after 24 hour culture (**Figure 2A**), suggesting that PGE_2_-induced decreases in *Sost* removed a suppressive effect of endogenous Wnt antagonists upon osteoblast function. Dickkopf1 (*Dkk1*) inhibits Wnt signaling in the same manner as does Sclerostin [42]. Whereas 50 nM–5 µM PGE_2_ dramatically reduced *Sost* transcript and protein (*not shown*) levels, PGE_2_ had no effect on *Dkk1* transcript (**Figure 2B**) nor its protein (**Figure 2C**) expression, suggesting that *Sost* repression is the primary mechanism of enhanced Wnt signaling in response to PGE_2_ treatment.

### PGE_2_ decreases Sost through cAMP-dependent mechanisms

**Figure 3:**
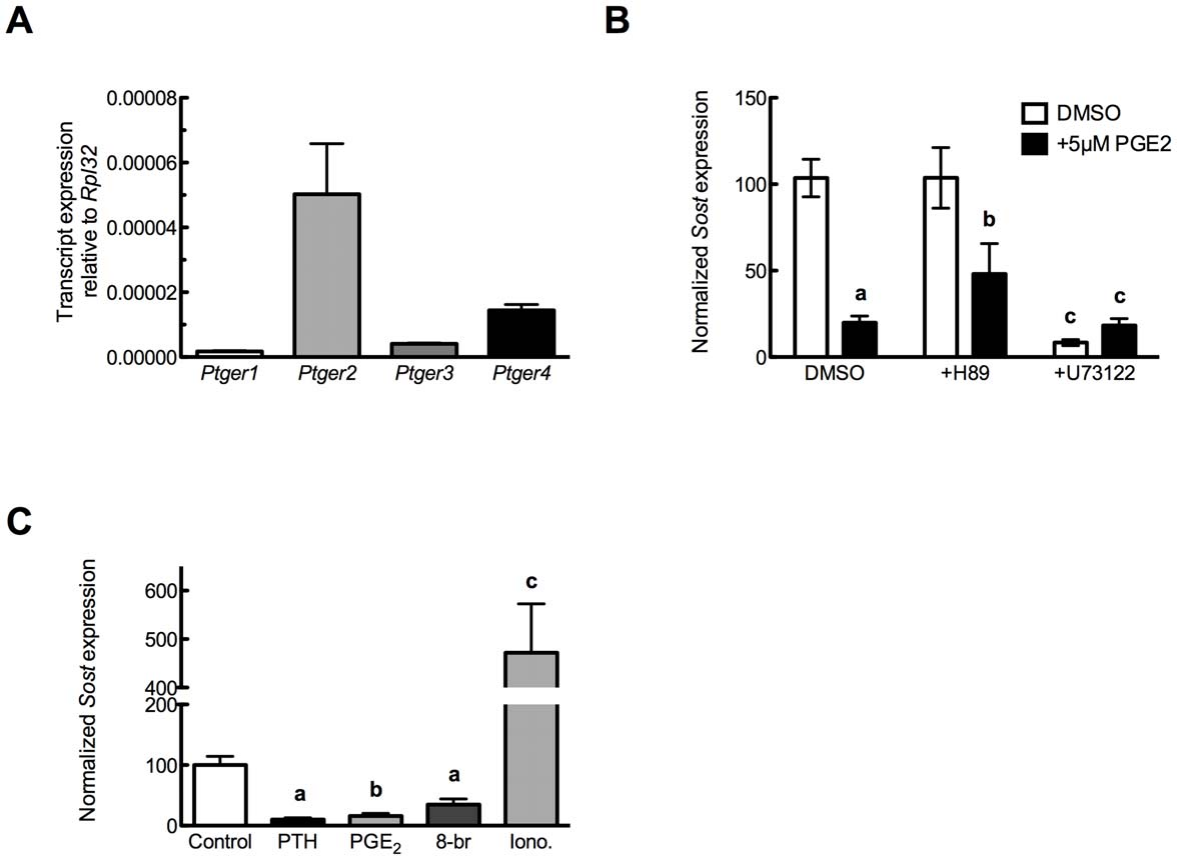
PGE_2_ receptor expression and influence of PGE_2_ selective agonists upon *Sost* expression. (**A**) UMR106.01 cells analyzed for *Ptger1*, *Ptger2*, *Ptger3*, and *Ptger4* transcript expression by qPCR. Data are normalized to *Rpl32*. n = 4 samples. (**B**) UMR106.01 cells were cultured with DMSO as vehicle control, 100 nM hPTH(1–34), 5 µM PGE_2_ in the presence and absence of inhibitors of protein kinase A (H-89, 2.5 µM) or phospholipase C (U73122, 10 µM), for 3 hours. Total RNA was analyzed for *Sost* and normalized to *Rpl32*. n = 4–8 samples. Compared to solvent control, **a** indicates *p*<.001 and **b** indicates *p*<0.05; **c** indicates *p*<.001. (**C**) UMR106.01 cells were cultured with DMSO as vehicle control, 100 nM hPTH(1–34), the cell-permeant cyclic AMP analogue 8-br-cAMP (1 mM) or the calcium ionophore ionomycin (1.3 µM) for 3 hours, after which total RNA was collected and analyzed for *Sost* and *Rpl32*. n = 4–8 samples. Versus Control, **a** indicates *p*<0.05, **b** indicates *p*<0.01, and **c** indicates *p*<0.001.

UMR 106.01 cells express all four classes of PGE_2_ receptors (EP1–EP4, encoded by *Ptger1*–*Ptger4*; **Figure 3A**), which are linked to the synthesis or mobilization of cAMP and Ca^2+^_i_. EP2 and EP4 increase cAMP levels, while EP1 increases Ca^2+^_i_ through a PLC-dependent mechanism; EP3 increases Ca^2+^_i_ and decreases cAMP [52]. To define which receptor(s) are responsible for mediating the suppressive effects of PGE_2_ upon *Sost*, UMR 106.01 cells were treated with 5 µM PGE_2_ in the presence of antagonists of protein kinase A (H-89, 2.5 µM) or phospolipase C (U73122, 10 µM) for 3 hours, after which time total RNA was collected and analyzed for *Sost* levels. In the absence of PGE_2_, inhibition of PLC/IP_3_/Ca^2+^_i_ signaling decreased basal *Sost* levels (**Figure 3B**), suggesting that release of intracellular calcium is important for maintaining *Sost* expression. In the presence of PGE_2_, the addition of H-89 appeared to attenuate PGE_2_-induced *Sost* suppression (**Figure 3B**) although this did not reach statistical significance (*p*<0.1 versus 5 µM PGE_2_ alone). In contrast, the addition of PGE_2_ to U73122-treated cells demonstrated no change compared to U73122 alone. The role of cAMP and Ca^2+^_i_ mobilization in suppressing *Sost* was tested using selective agonists. UMR106.01 cells treated with the cAMP mimetic 8-bromo-cAMP (1 mM) demonstrated similar suppression of *Sost* as 5 µM PGE_2_-treated cells (**Figure 3C**), whereas 1.3 µM ionomycin treatment significantly increased *Sost* expression. These data indicate that PGE_2_ receptors linked to increased cAMP—*Ptger2* or *Ptger4*—are involved in the capacity for PGE_2_ to decrease *Sost*.

**Figure 4:**
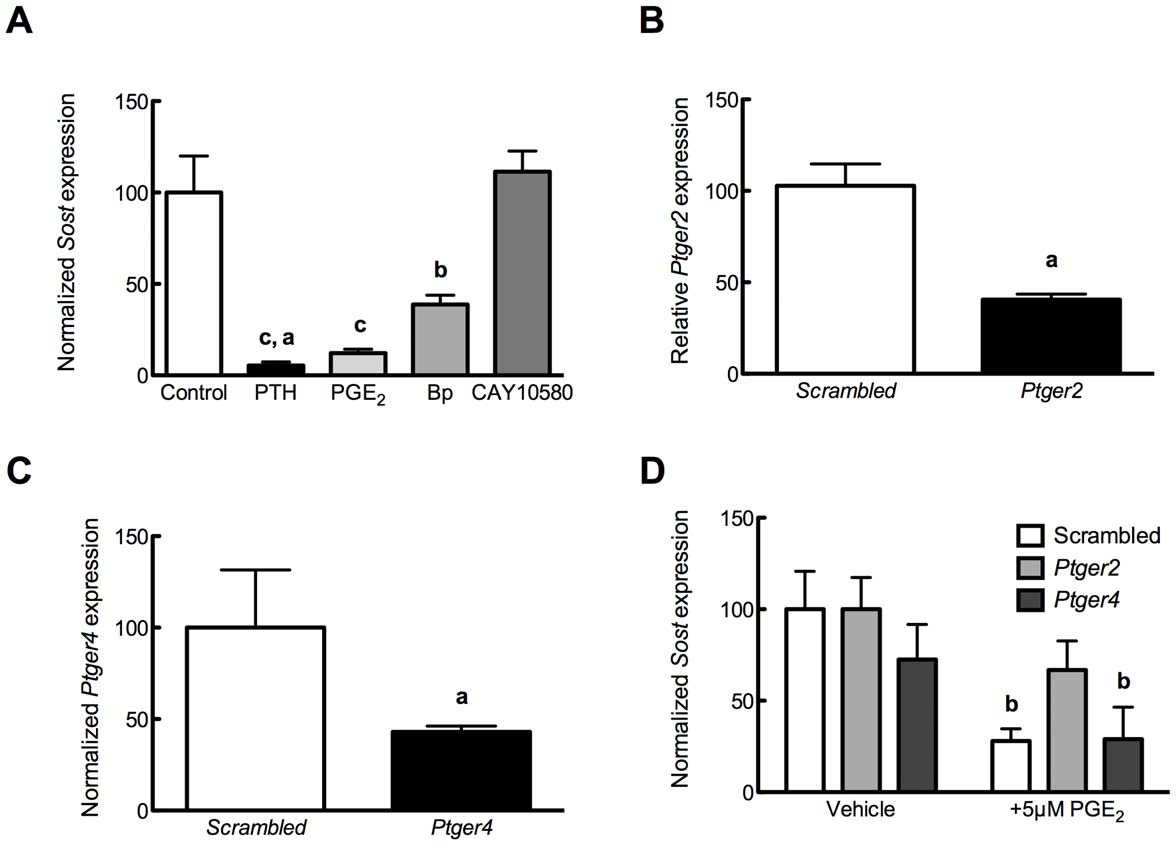
PGE_2_ signals through *Ptger2* to decrease *Sost* expression. (**A**) Cells were cultured for 3 hours in the presence of PTH (100 nM), PGE_2_, EP2 agonist butaprost, or EP4 agonist CAY10580 (each 500 nM). *Sost* expression was analyzed by qPCR and normalized to *Rpl32*. n = 5 samples. Compared to vehicle control, **b** indicates *p*<0.01 and **c** indicates *p*<0.001; **a** indicates *p*<0.01 versus CAY10580. (**B**) UMR106.01 cells were cultured with 50 nM of scrambled or *Ptger2* siRNA for 48 hours, after which *Ptger2* expression was examined by qPCR. n = 4 samples. Compared to vehicle control, **a** indicates *p*<0.05. (**C**) UMR106.01 cells were cultured with 50 nM of scrambled or *Ptger4* siRNA for 48 hours, after which *Ptger4* expression was examined by qPCR. n = 4 samples. Compared to vehicle control, **a** indicates *p*<0.05. (**D**) UMR106.01 cells were cultured with 50 nM of scrambled, *Ptger2*, or *Ptger4* siRNA for 48 hours, then with 5 µM PGE_2_ for 3 hours, after which time total RNA was collected and analyzed for *Sost* and *Rpl32*. n = 5 samples. Compared to vehicle control, **b** indicates *p*<0.01.

Specific agonists for EP2 (butaprost, [53]) or EP4 (CAY10580, [54]) were also tested for their ability to mimic the suppressive effects of PGE_2_ on *Sost* transcription. Butaprost mimicked the ability of PGE_2_ to decrease *Sost*, whereas CAY10580 had no effect on *Sost* levels (**Figure 4A**). siRNA directed against *Ptger2* (**Figure 4B**) or *Ptger4* (**Figure 4C**) reproducibly decreased target transcript expression by 60% relative to non-silencing, scrambled siRNAs. Knock-down of *Ptger2*, but not *Ptger4*, significantly impaired the ability of PGE_2_ to suppress *Sost* expression (**Figure 4D**), indicating the requirement for the *Ptger2* receptor for PGE_2_-specific activation of Wnt signaling.

### Cycloheximide, but not Actinomycin D, influences PGE_2_ suppression of Sclerostin

**Figure 5:**
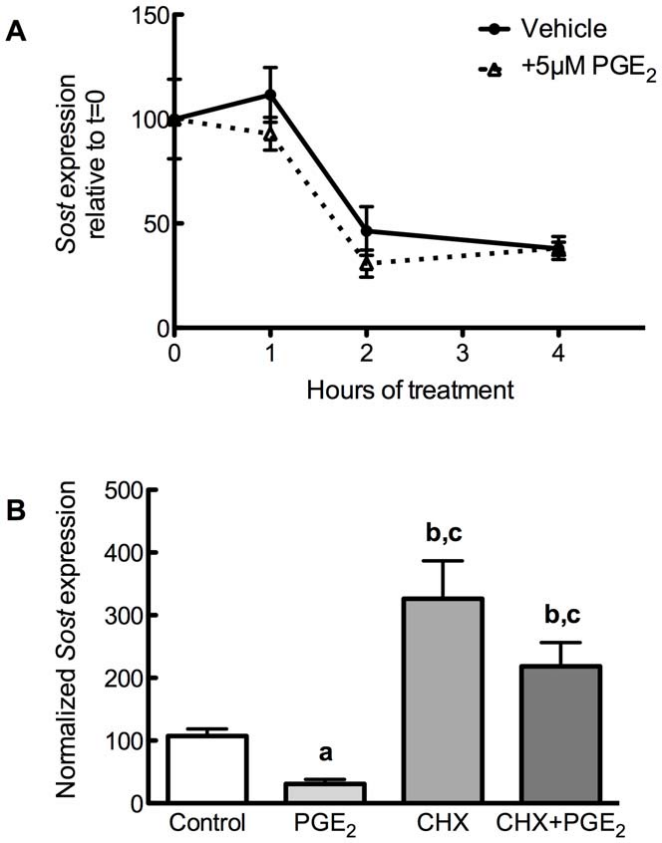
Effects of actinomycin D or cycloheximide upon PGE_2_ suppression of Sclerostin. (**A**) UMR106.01 cells were serum-starved for 1 hour, treated with 2.5 µg/mL actinomycin D with or without 5 µM PGE_2_, and collected 3 hours later. cDNA was prepared for qPCR analysis of *Sost* and *Rpl32*. n = 4 samples. (**B**) UMR 106.01 cells were treated with combinations of 10 µg/mL cycloheximide and 5 µM PGE_2_ for 3 hours. Samples were analyzed by qPCR for *Sost* and *Rpl32*. n = 4 samples. Compared to vehicle control, **a** indicates *p*<0.05 and **b** indicates *p*<0.001; compared to 5 µM PGE_2_, **c** indicates *p*<0.001.

We examined the transcriptional and translational mechanisms whereby PGE_2_ regulates *Sost* expression. UMR106.01 cells were treated with 5 µM PGE_2_ for 3 hours in the presence or absence of the RNA polymerase II inhibitor Actinomycin D (2.5 µg/mL), after which time total RNA was collected and analyzed. The suppressive influence of PGE_2_ on *Sost* transcription was consistent in cells treated with or without actinomycin D (**Figure 5A**), indicating that PGE_2_ does not promote the degradation of *Sost* transcript.

Next, UMR106.01 cells were treated with or without 5 µM PGE_2_ for 3 hours in the presence or absence of the protein translation inhibitor cycloheximide (CHX; 10 µg/mL). In the absence of PGE_2_, CHX increased *Sost* transcript (**Figure 5B**); in cells treated with PGE_2_ and CHX, there was no suppressive effect of PGE_2_ upon *Sost* expression, indicating that PGE_2_ requires *de novo* protein synthesis in order to decrease *Sost*. Similar results were observed after 1 hour of CHX or PGE^2^ treatment (*data not shown*).

### Transcriptional regulation of Sost by PGE_2_ does not involve Mef2 or BMPs

**Figure 6:**
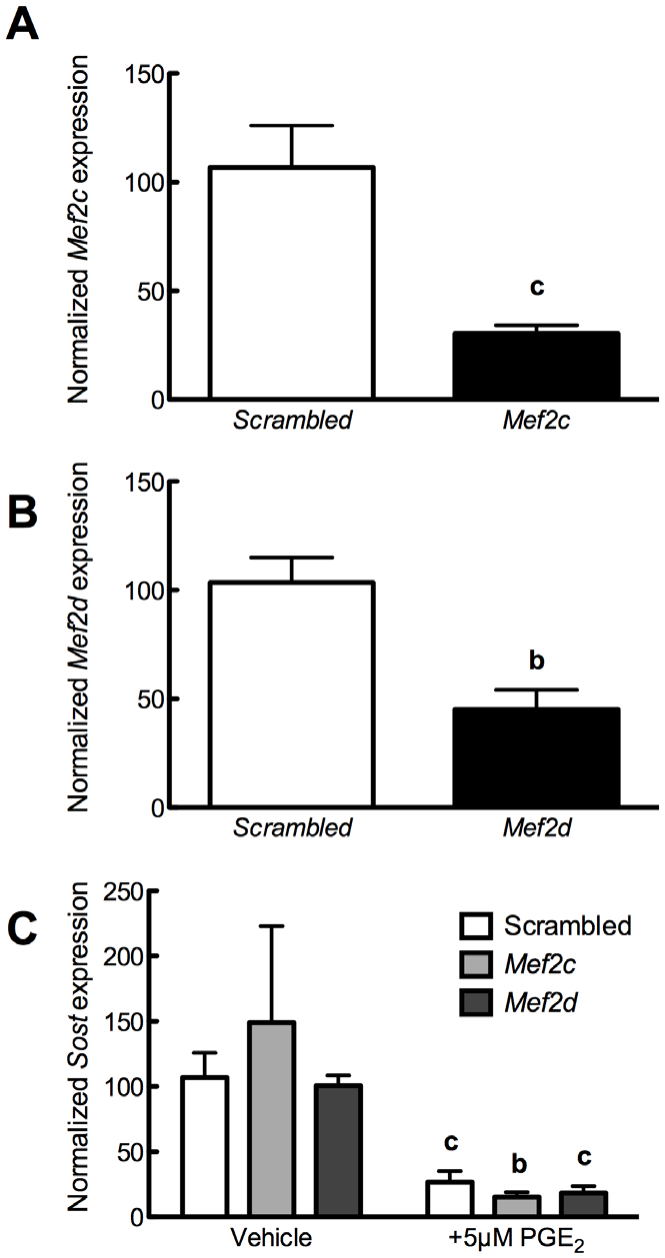
Reductions in MEF2 expression do not impair PGE_2_ decrease of *Sost*. (**A**) UMR106.01 cells were cultured with 50 nM of scrambled or *Ptger4* siRNA for 48 hours, after which *Mef2c* expression was examined by qPCR. n = 4 samples. Compared to scrambled control, **c** indicates *p*<0.001. (**B**) UMR106.01 cells were cultured with 50 nM of scrambled or *Ptger4* siRNA for 48 hours, after which *Mef2d* expression was examined by qPCR. n = 4 samples. Compared to scrambled control, **c** indicates *p*<0.01. (**C**) UMR106.01 cells were cultured with 50 nM of scrambled, *Mef2c*, or *Mef2d* siRNA for 48 hours, then with 5 µM PGE_2_ for 3 hours, after which time total RNA was collected and analyzed for *Sost* and *Rpl32*. n = 5 samples. Compared to target siRNA control, **b** indicates *p*<0.01 and **c** indicates *p*<0.001.

We have previously demonstrated that the MEF2 family of transcription factors are responsible for sensitizing the *Sost* distal enhancer ECR5 to PTH [45]. We employed siRNA against *Mef2c* or *Mef2d* in order to determine whether these transcription factors are involved in the capacity for PGE_2_ to decrease *Sost*. 48 hours after transfection, expression of *Mef2c* (**Figure 6A**) and *Mef2d* (**Figure 6B**) was reduced approximately 70% and 55%, respectively, compared to scrambled siRNA controls. Knock-down of *Mef2c* or *Mef2d* did not alter the ability of 5 µM PGE_2_ to decrease *Sost* transcript (**Figure 6C**), suggesting that PGE_2_ does not decrease *Sost* expression by disrupting Mef2 activity.

**Figure 7:**
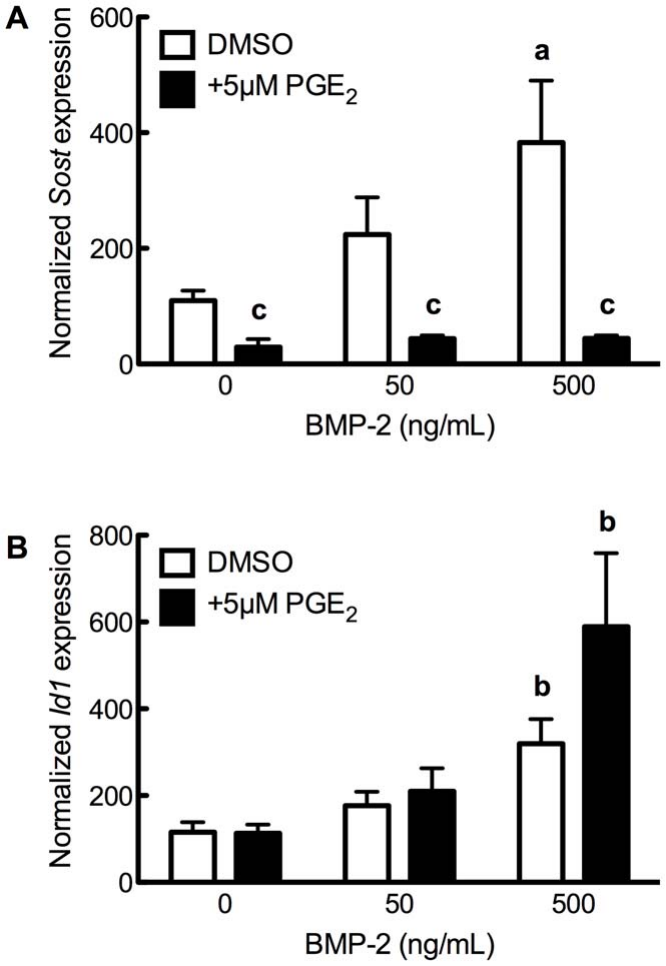
PGE_2_ decreases *Sost* without affecting BMP signaling. (**A**) Cells were treated with BMP-2 (0–500 ng/mL) in the presence or absence of 5 µM PGE_2_ for 3 hours, after which (**A**) *Sost* or (**B**) *Id1* expression was monitored. Compared to vehicle control, **a** indicates *p*<0.05 and **b** indicates *p*<0.01; compared to BMP-2 without PGE_2_; , **c** indicates *p*<0.001.

We, and others, have previously demonstrated the transcriptional influence of bone morphogenetic proteins on *Sost*: exogenous BMPs or constitutively-active BMP receptor 1A increase *Sost* expression [50], [51], [55], whereas dominant-negative BMP Receptor 1A decreases *Sost* transcription [55]. To examine whether PGE_2_ signaling disrupted BMP induction of *Sost*, UMR106.01 cells co-cultured with 5 µM PGE_2_ and BMP-2 (0–500 ng/mL) for 3 hours. BMP-2-treated cells increased *Sost* expression (**Figure 7A**), whereas co-culture with PGE_2_ prevented *Sost* induction. BMP-2 increased *Id1*, a direct Smad target gene, independent of PGE_2_ (**Figure 7B**), indicating that BMP signaling was unaffected by PGE_2_ treatment. These data suggest that PGE_2_ decreases *Sost* transcription independent of the BMP signaling, and hence the effect of PGE2 upon *Sost* is downstream of BMPs.

### PTH does not require prostaglandins to decrease Sost

**Figure 8:**
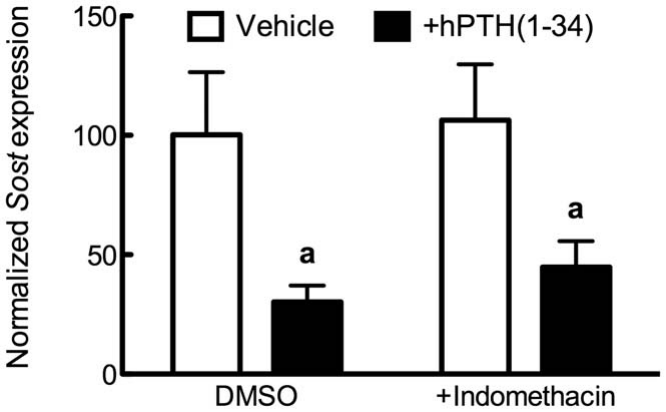
PTH decreases *Sost* expression independent of prostaglandins. Cells were exposed to 1 µM indomethacin for 24 hours, then treated with 100 nM hPTH(1–34) or vehicle control for 24 further hours. *Sost* expression was analyzed by qPCR and normalized to *Rpl32*. Compared to vehicle or indomethacin control, **a** indicates *p*<0.05.

PTH increases COX-2 expression and subsequent synthesis and release of prostaglandins [56], . Because both PTH and PGE_2_ decrease *Sost* transcription through cAMP/PKA mechanisms, we next examined whether the capacity for PTH to decrease *Sost* required prostaglandins. Cells were treated for 24 hours in reduced serum (2%) conditions in the presence of 0.05% DMSO or 1 µM indomethacin; thereafter, a subset of cells were treated for 24 hours in the presence of 100 nM hPTH(1–34). As shown in **Figure 8**, there was a similar decrease in *Sost* expression in cells treated with PTH with or without indomethacin co-treatment. Thus, PTH does not require prostaglandins to decrease *Sost* transcription, suggesting that PTH and PGE_2_ function through independent, parallel pathways that converge upstream of Sclerostin's transcriptional regulation.

## Discussion

Sclerostin is a robust inhibitor of bone formation, such that its absence leads to increased bone formation, and in high amounts it causes bone loss. Thus, regulation of its expression, as well as that of other potent skeletally anabolic or catabolic proteins, is under intensive investigation as a therapy for those afflicted with osteoporosis, or as a means of hastening fracture repair. Indeed, a monoclonal antibody which inhibits Sclerostin function has already been shown to increase bone formation and strength in ovariectomized rats beyond that of non-ovariectomized controls [58] and in aged male rats [59]. Despite its clinical importance in an aging population, the molecular mechanisms controlling Sclerostin expression are only beginning to be unraveled [43], [45]. Within this work, we demonstrate that PGE_2_, a paracrine signaling agent with diverse effects on skeletal homeostasis, decreases *Sost* transcription through the EP2 receptor subclass (encoded by *Ptger2*). Reductions in *Sost* transcription by PGE_2_ was shown to involve cAMP and PKA, *de novo* protein synthesis, and to occur independently of BMP or MEF2 signaling.

### PGE_2_ decreases Sost expression via PKA and Ptger2

We observed rapid suppression of *Sost* by PGE_2_ between 50 nM–5 µM, with a calculated IC_50_ of 41 nM (*data not shown*). Reductions in *Sost* transcript in response to PGE_2_ were rapid, occurring within 1 hr of PGE_2_ addition, and were sustained, remaining at 30% expression compared to vehicle-treated samples after 24 hours of culture. These results are similar to *in vivo* calvarial and *in vitro* cell culture models treated with PTH [43], as well as murine models of constitutively-active PTHR1 receptor [44], [60].

Osteoblastic cells express all *Ptger* receptor genes [61], [62], suggesting that PGE_2_ can exert biological effects through both cAMP and Ca^2+^_i_ signaling pathways. Much of the anabolic effect of PGE_2_ is mediated through cAMP *via* EP2 and EP4 [63]. The cAMP analogue 8-bromo-cAMP mimicked the effect of PGE_2_ upon *Sost* transcription. Inhibition of PLC/IP_3_ with U73122 did not prevent PGE_2_ from decreasing *Sost*, indicating that this pathway is not obligate. Interestingly, the calcium ionophore, ionomycin, significantly increased *Sost* transcription nearly 5-fold over vehicle controls after 3 hours of treatment. This would suggest stimulation of MEF2 transcriptional activity in response to increased Ca^2+^_i_, as has been shown in skeletal muscle fibers [64], [65]. Keller and Kneissel have shown a modest decrease in *Sost* transcription in response to a similar dose of ionomycin [43], but they measured *Sost* levels after 24 hours of ionomycin treatment (rather than 3 hours, as in the study herein). Whether these contrasting results are due to timing of ionomycin treatment, or are secondary to prolonged cellular stress due to supra-physiologic Ca^2+^_i_ [66], remains to be elucidated.

The requirement for cAMP/PKA to decrease *Sost* thus implicated either EP2 or EP4 receptor. Butaprost, a selective agonist for EP2 [67], decreased *Sost* transcription by 70%, whereas CAY10580, an EP4 agonist [54], had no significant effect upon *Sost* levels. The EP2 receptor was further implicated in mediating the suppressive effects of PGE_2_, as siRNA directed against *Ptger2*, but not scrambled or *Ptger4* siRNA, prevented PGE_2_-induced decreases in *Sost*. In total, these data indicate that PGE_2_ signals through EP2 to decrease *Sost* expression.

### PGE_2_ and PTH decrease Sost through parallel pathways

PTH has also been shown to increase COX-2 expression and PG release [56], [57]. Because PTH and PGE_2_ are both capable of mobilizing the same second messengers (cAMP and IP_3_), and because both PTH and PGE_2_ decreased *Sost* transcription through similar mechanisms involving cAMP and MEF2, we examined whether PTH required PGE_2_ (or other PGs) in order to decrease *Sost*. Inhibition of COX-1 and COX-2 function with 1 µM indomethacin increased *Sost* expression (*data not shown*), indicating tonic suppression of *Sost* by endogenously-produced prostaglandins. Cells treated with both PTH and indomethacin continued to demonstrate suppression of *Sost*, indicating that PTH does not require prostaglandins to decrease *Sost* levels in mature osteoblastic cells.

There are several distinctions that must be made regarding *Sost* regulation by PTH and PGE_2_. Keller and Kneissel [43] demonstrated that PTH rapidly decreases *Sost* expression in UMR106.01 cells through a cyclic AMP-dependent pathway. Leupin *et al.* identified the MEF2 family of transcription factors as a requirement for driving bone-specific *Sost* expression, and as a target of PTH [45]. Within, we demonstrate that PGE_2_, like PTH, decreases *Sost* in a cyclic AMP-dependent pathway. While PTH decreases *Sost* expression through unknown interactions with MEF2C and MEF2D, we continued to observe *Sost* suppression in cells transfected with *Mef2c* or *Mef2d* siRNA, demonstrating one key difference between PGE_2_ and PTH. Similarly, inhibition of *de novo* protein synthesis with cycloheximide maintains PTH suppression of *Sost* [43], whereas cycloheximide prevented PGE_2_ reductions in *Sost*. These data indicate that, while both PGE_2_ and PTH use cAMP to decrease *Sost*, there is divergence downstream from cAMP/PKA in the signaling pathways utilized by PTH or PGE_2_ for *Sost* suppression.

Current FDA-approved therapies for combating osteoporosis are limited to drugs, like bisphosphonates, that inhibit bone resorption. Intermittent PTH is the only FDA-approved therapy that promotes bone formation, although treatment is currently limited to 18 months. The sclerosing bone dysplasias sclerosteosis and van Buchem disease are caused by decreased or absent Sclerostin expression, and thereby implicate Sclerostin as a very potent inhibitor of bone formation. Thus, mechanisms for manipulating *Sost* expression may likely provide a powerful means of increasing bone mass. Within, we have elucidated a novel mechanism of Sclerostin regulation. Continued efforts to modulate its expression and/or activity will likely allow for novel anabolic agents for conditions of bone loss.

## Materials and Methods

***Cell culture-*** UMR106.01 cells, which express phenotypic markers of mature osteoblasts [68], were cultured in MEM with Earle's Salts (Invitrogen) supplemented with 10% fetal bovine serum (FBS; Invitrogen) and 1% penicillin and streptomycin (P&S; Invitrogen). Cells were routinely sub-cultured, using 0.05% trypsin/EDTA when 80–90% confluent; for experiments, cells were seeded into 35 mm^2^ dishes at 5 k/cm^2^, and experiments were performed two days thereafter.

***Chemicals and reagents-*** PGE_2_ and PGF_2α_ (Cayman Chemical) were dissolved in DMSO as stock concentrations of 10 mM. Human PTH(1–34) (Bachem) was dissolved in HBSS+0.1% BSA and stored at 100 µM aliquots. H-89 or 8-br-cAMP (EMD Biosciences) were dissolved in sterile water; U73122, ionomycin (both EMD Biosciences), butaprost, or CAY10580 (both Cayman Chemical) were dissolved in DMSO.

***Quantitative PCR-*** At the indicated time, cells were washed with PBS and total RNA was collected using RNeasy Mini kit (Qiagen). Total RNA (200–1000 ng) was reverse-transcribed with QuantiTect Reverse Transcription Kit (Qiagen), which includes a genomic DNA elimination step. qPCR was performed using QuantiFast Probe PCR Kit (Qiagen) on a Mastercycler® realplex2 (Eppendorf). Proprietary primer and TaqMan probe sets were purchased from Applied Biosystems. Amplification conditions were 95°C for 3 minutes, followed by 40 cycles at 95°C for 3 seconds and 60°C for 30 seconds. Quantitative PCR results were normalized to loading control (*Rpl32* or *Tbp*) transcript level to yield ΔC_t_, then normalized to control conditions to generate ΔΔC_t_. Relative or fold change in expression was subsequently calculated using the formula 2^−ΔCt^ or 2^−ΔΔCt^, as described in [69].

***Dkk1 ELISA***- For measurement of Dkk1 protein production, cells were prepared as described above and cultured for 24 hours in 0.05% DMSO or 5 µM PGE_2_. Conditioned media and whole cell protein lysates were collected and frozen at −20 C until analysis. Dkk1 protein levels in conditioned media were analyzed using a commercially available ELISA against murine Dkk1 (R & D Systems), and results were normalized to whole cell protein concentration.

***siRNA*** **-** small, interfering RNA against *Ptger2*, *Ptger4*, *Mef2c*, and *Mef2d* was purchased from Qiagen, as was scrambled, non-silencing control. Cells were seeded at a density of 40,000 cells *per* well in a 24-well plate in media supplemented with 10% FBS and 1% P/S. 30 minutes thereafter, 50 nM scrambled, non-silencing or *Ptger2* was prepared with HiperFect (Qiagen) in 100 µL of serum- and antibiotic-free media. 20 minutes later, siRNA/HiperFect/media was overlayed on top of the cells, which were returned to the incubator. Experiments were performed 48 hours later.

***Statistical analysis-*** Each data set was acquired a minimum of three times, in duplicate. qPCR data were first analyzed relative to the internal control *Rpl32*, then normalized to vehicle control, in order to minimize inter-experimental variation. Results are expressed as mean±standard error of the mean. Data were analyzed by Kruskal–Wallis or ANOVA followed by Dunnet or Tukey post-hoc tests where appropriate. *p*<0.05 was considered statistically significant.
